# There is only one big risk you should avoid at all costs, and that is the risk of doing nothing

**DOI:** 10.1007/s12471-015-0675-8

**Published:** 2015-03-12

**Authors:** J. Daemen

**Affiliations:** Division of Interventional Cardiology, Department of Cardiology, Thoraxcenter, Erasmus University Medical Center, Room Ba593, ’s Gravendijkwal 230, PO Box 2040, 3000 CA Rotterdam, The Netherlands

This issue of the *Netherland Heart Journal* presents the meta-analysis of Rasoul et al. [1] in which they question the optimal treatment strategy for patients presenting with ST-segment elevation myocardial infarction (STEMI) and multivessel coronary artery disease, an extremely relevant question to those who are regularly confronted with STEMI patients. The study queues up in line with a large number of studies and meta-analyses on the same topic published in the past 10 years. Unfortunately, their results are conflicting, which is not surprising given the differential study designs and potpourri of confounders in the research question.

Patients who are diagnosed with multivessel disease fair worse than those with single-vessel disease as reflected by higher morbidity and mortality rates, a fact that holds irrespective of the patient’s clinical status at presentation [2–4]. The latter triggered the discussion on how to achieve the most optimal, and complete, revascularisation strategy by either multivessel percutaneous coronary revascularisation (PCI) or coronary artery bypass graft surgery in which besides multiple clinical factors angiographic characteristics such as lesion complexity and haemodynamic consequence as determined by fractional flow reserve (FFR) measurements come into play [5, 6]. The latter proved to be of particular importance because the capacity of even the most experienced interventional cardiologists in assessing lesion severity and haemodynamic consequences proved to be limited. In the setting of primary PCI, assessing lesion severity becomes even more challenging, especially when there is concomitant haemodynamic instability and/or concurrent infusion with vasoactive substances. Subsequently, when the operator is convinced that a particular non-culprit lesion has to be treated, the following dilemma already pops up. A decision has to be made on direct versus staged treatment or postponing the potential intervention awaiting residual symptoms or objective evidence of ischaemia. Comparing ‘ad hoc’ multivessel versus infarct-related artery-only revascularisation in the setting of STEMI and multivessel disease is therefore an oversimplification of the actual clinical problem.

Clear answers can only be provided by large dedicated randomised controlled trials with adequate power and predefined guiding on post-procedural care, patient information and treatment timing of repeat interventions. The lack of these clear guidelines, typically applied in randomised controlled trials, is exactly the reason why observational and mostly retrospective studies fail to provide convincing answers to our clinical dilemma. The latter is demonstrated by clear differences in the cohorts of patients in individual observational studies. Treatment groups differ in age, diabetes, presence of haemodynamic instability and disease complexity. Not even to speak about the huge imbalance in numbers of patients per group; multivessel PCI was performed in only 13.9 % of the patients versus 86.1 % who received infarct artery only treatment as demonstrated by Rasoul and colleagues. Pooling data makes things even more complicated because this potpourri of studies is characterised by differential inclusion criteria, follow-up durations, endpoint definitions and most importantly the definition of multivessel versus infarct-related artery-only PCI. Therefore, if the ‘raw material’ is flawed, the conclusions of systematic reviews and meta-analyses cannot be trusted [7].

Preventive Angioplasty in Acute Myocardial Infarction (PRAMI) was the first randomised controlled trial showing a benefit for complete revascularisation, demonstrating a 65 % reduction in major adverse cardiac events over a mean follow-up of 23 months for total versus culprit-only PCI [8]. The PRAMI was followed by the recently presented the Complete Versus Lesion-only PRimary PCI Trial (CvLPRIT) study, a prospective open-label, multicentre UK trial including 296 patients (excluding shock patients). The study confirmed that major adverse cardiac events rates were significantly lower in those who received multivessel revascularisation, driven by a reduction in all-cause mortality, myocardial infarction, heart failure and repeat PCI. However, in both studies, staging the intended multivessel revascularisation was allowed. In CvLPRIT, more than 27 % of the patients included received non-culprit artery treatment several days after the primary PCI. Although these two important clinical trials shed new light on our research question, they also triggered a similar amount of debate. Both trials were open-label, and patients were aware of the presence of residual stenosis which might have triggered repeat events. As mentioned earlier in the text, staged PCI was discouraged, but still happened in a considerable number of patients. No data on left ventricular function were available, and finally, most studies concerning revascularisation have defined significant stenosis as a narrowing of at least 70 %, while in PRAMI, lesions with a stenosis of more than 50 % were eligible for revascularisation.

Besides the wealth of studies on the topic, one can only conclude that a simple yes or no answer to the question whether to perform ad hoc multivessel PCI in STEMI patients with multivessel disease simply does not exist today and probably will not even exist in the future. Therefore, the best we can do is trust on our own personal judgment of the individual patient, keeping in mind the following facts that we do know:

- Assessing non-culprit lesions in the setting of a primary PCI is challenging because lesion severity can often be overestimated.
- Staging allows haemodynamic lesion assessment using fractional flow reserve, which proved to result in avoiding unnecessary interventions, saving costs and improving hard clinical endpoints.
- Stenting in a prothrombotic inflammatory milieu is not preferred.
- Unforeseen peri-procedural complications are always at risk.
- Direct or staged multivessel PCI decreases the need for further hospitalisations and subsequent interventions that might be cost saving.
- Individual non-culprit lesion complexity, procedure time, contrast volume and haemodynamic status of the patient should be taken into account at all times.
- In case of complex multivessel disease, surgical management during the acute and subacute phase proved to be associated with excellent 30-day and 1-year survival [9].

## References

[1] Rasoul S, van Ommen V, Vainer J, et al. Multivessel revascularisation versus infarct-related artery only revascularisation during the index primary PCI in STEMI patients with multivessel disease: a meta-analysis. Neth Heart J. 2015;23. doi: 10.1007/s12471-015-0673-x. 10.1007/s12471-015-0674-9 PMC4368524 25884095

[2] Parodi G, Memisha G, Valenti R (2005). Five year outcome after primary coronary intervention for acute ST elevation myocardial infarction: results from a single centre experience. Heart.

[3] Rosner GF, Kirtane AJ, Genereux P (2012). Impact of the presence and extent of incomplete angiographic revascularization after percutaneous coronary intervention in acute coronary syndromes: the Acute Catheterization and Urgent Intervention Triage Strategy (ACUITY) trial. Circulation.

[4] Hannan EL, Wu C, Walford G (2009). Incomplete revascularization in the era of drug-eluting stents: impact on adverse outcomes. JACC Cardiovasc Interv.

[5] Daemen J, Boersma E, Flather M (2008). Long-term safety and efficacy of percutaneous coronary intervention with stenting and coronary artery bypass surgery for multivessel coronary artery disease: a meta-analysis with 5-year patient-level data from the ARTS, ERACI-II, MASS-II, and SoS trials. Circulation.

[6] Tonino PA, De Bruyne B, Pijls NH (2009). Fractional flow reserve versus angiography for guiding percutaneous coronary intervention. N Engl J Med.

[7] da Costa BR, Juni P (2014). Systematic reviews and meta-analyses of randomized trials: principles and pitfalls. Eur Heart J.

[8] Wald DS, Morris JK, Wald NJ (2013). Randomized trial of preventive angioplasty in myocardial infarction. N Engl J Med.

[9] Gu YL, van der Horst IC, Douglas YL, Svilaas T, Mariani MA, Zijlstra F (2010). Role of coronary artery bypass grafting during the acute and subacute phase of ST-elevation myocardial infarction. Neth Heart J.

